# Providing AI expertise as an infrastructure in academia

**DOI:** 10.1016/j.patter.2023.100819

**Published:** 2023-08-11

**Authors:** Marie Piraud, Andrés Camero, Markus Götz, Stefan Kesselheim, Peter Steinbach, Tobias Weigel

**Affiliations:** 1 Helmholtz AI, Germany; 2 Helmholtz Munich, Neuherberg, Germany; 3 Karlsruhe Insitute of Technology, Eggenstein-Leopoldshafen, Germany; 4 Hereon, Geesthacht, Germany; 5 German Aerospace Center, Wessling, Germany; 6 Forschungszentrum Jülich, Jülich, Germany; 7 Helmholtz-Zentrum Dresden-Rossendorf, Dresden, Germany; 8 German Climate Computing Center, Hamburg, Germany

## Abstract

Artificial intelligence (AI) is proliferating and developing faster than any domain scientist can adapt. To support the scientific enterprise in the Helmholtz association, a network of AI specialists has been set up to disseminate AI expertise as an infrastructure among domain scientists. As this effort exposes an evolutionary step in science organization in Germany, this article aspires to describe our setup, goals, and motivations. We comment on past experiences, current developments, and future ideas as we bring our expertise as an infrastructure closer to scientists across our organization. We hope that this offers a brief yet insightful view of our activities as well as inspiration for other science organizations.

## Main text

### Introduction

Picture Anna, an experimental physicist, who is under increasing pressure to use artificial intelligence (AI) to analyze the data she generates during her research. She has access to trainings and high-performance computing infrastructure. Due to the rapid evolution of the field, the gap grew too large for her to use AI in practice. This applies to many of the 23,000 scientists of the Helmholtz association, who operate in fundamental and natural sciences, as well as engineering. To boost its academic research system, the Helmholtz association therefore launched Helmholtz AI, a Germany-wide platform that provides AI expertise as an infrastructure in a collaborative fashion. Key motivations behind this effort are to increase the productivity of Anna and broaden her competitiveness in the academic system, as well as reduce the turnaround of insights. Last but not least, this can be seen as a step toward reducing the gap between academic and industrial research using or on machine learning.

### Helmholtz AI consulting at a glance

**Figure 1:**
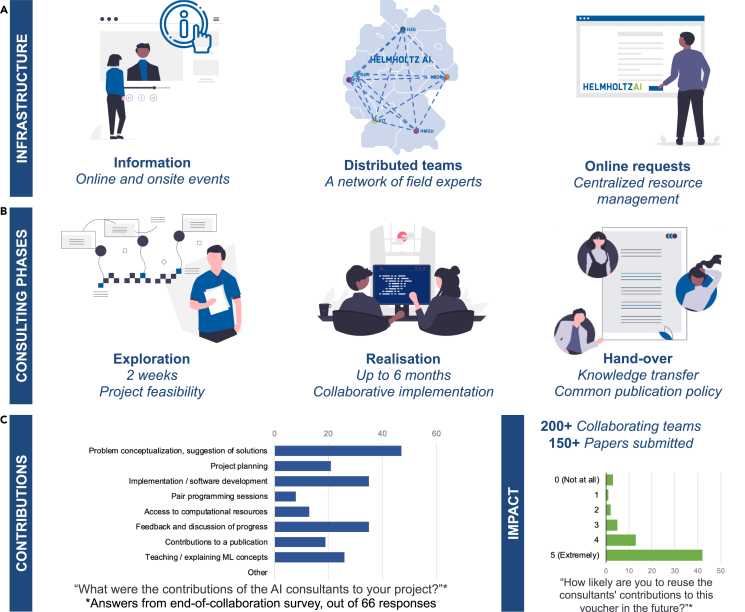
Helmholtz AI consulting in a nutshell (A) Main features of the Helmholtz AI consulting infrastructure. (B) Consulting phases. (C) Contributions and impact in a nutshell.

AI consultants are method specialists, distributed in teams across Germany with a specific field of expertise (Figure 1A). They work collaboratively with researchers on short- to mid-term research questions. Collaborators can apply for consulting through an online system, which helps to perform quality control and match demand with capabilities and capacity.

Approved requests initiate a 2-week to 6-month collaboration following pre-defined phases (Figure 1B). This may involve one or more AI consultants at no cost to collaborating researchers. The match is made according to research fields but also based on methodological expertise and specific experience, e.g., handling certain types of data like images or time series.

The focus of the collaboration is on knowledge transfer and enablement of the scientists to use AI in practice. Consultants help in the proper choice of methods and tools, as well as a first implementation in order to close the gap mentioned above.

After 3 years of implementation, 35 FTE (full-time equivalent) AI consultant positions have been filled. Over 200 research teams from 18 institutions submitted more than 300 consulting requests that have been addressed. Impactwise, 98% of collaborators state in a survey that they are very likely to recommend collaborating with the consultants to a colleague and 91% that they are likely to reuse the consultants’ contributions in the future (Figure 1C). Furthermore, the consulting teams submitted more than 150 papers^1^^,^^2^^,^^3^^,^^4^^,^^5^^,^^6^ during that period. At least nine research grant proposals were submitted by collaborators based on the consultants’ work.

We, the consultant team leaders, have scrutinized this approach and outline our lessons learned for a wider audience here.

### Shaping the consulting offer

#### Reach out and engage

In the initial phase, virtual and onsite information events were organized to reach out and probe the needs of the community. In parallel, consulting started with pilot projects in the local institutions of the consultant teams. This revealed that expectations regarding the application of AI can range anywhere from extreme skepticism to expecting magic solutions.

The following communication strategies have proven useful to embark on fruitful collaborations:

• We communicate expectations modestly, e.g., restating that modern AI can only pick up what was provided (as data).
• Extensive discussions lead to early discovery of misconceptions.
• The targeted usage of structured processes with defined goals, deliverables, and tools supports realistic and actionable expectations.

Institutional barriers, and related fear of conflict of interest specific to our network setting, also came up as an obstacle to overcome with a positive communication culture. In general, we have observed that the communication process results in a positive experience with results that highly impact the research fields.

#### Establish an exploration phase

As our AI experts are usually no specialists in our collaborators’ domain, collaboration can require extensive setup time. Additionally, as understanding and expectations of AI varies among our collaborators, the submitted requests are rarely formulated as “data in, model out.”

To structure and formalize this, a three-phase process has been established (Figure 1B). First, a 2-week exploration phase takes place. Second, a longer collaboration, lasting up to 6 months, can be installed, which we refer to as realization phase, followed by the hand-over and reporting phase of the project.

The exploration phase aims at the following goals:

1. Obtain the required understanding of the concepts of the domain to understand the question.
2. Get acquainted with the available data.
3. Analyze the state-of-the-art. Formulate a first approach that can solve the question at hand and scope the realization phase.
4. Offer a natural and socially sustainable point to end the collaboration, for example in case of unmet expectations by both parties.

All aspects are essential to the overall process. First, scientists can experience challenges communicating their research question to a person from a different field. Second, getting access to data is indispensable to ensure full understanding of it. We have often observed that the quality, consistency, and quantity of data are often overestimated by collaborators. Finally, beyond expectation management, the active formulation of a first solution should validate the proper understanding of the question and typically stimulates further discussions on both sides.

#### Meet the needs of users

Our collaborators have varying levels of understanding of AI in general. Therefore, AI consultants need to carefully analyze requirements with respect to the life cycle of data-driven applications.^7^ We find that user needs are commonly aligned with the major development stages. The most frequent and recurring ones are:

• conceptual design
• data exploration and preparation
• model implementation and verification
• benchmarking and tuning
• exploitation

In scientific settings, the last point usually entails some form of productive service, deployment, or software application as well as a joint publication of the findings. In addition to producing these artifacts of daily data science and machine-learning operations, our consultants also receive requests to teach, co-supervise, or quality control AI-based research. To meet those needs, deliverables span classical research methodologies, like literature research or scientific discussion, agile software development—including pair-programming and code reviews—and the generation of material for written documents, such as plots and visualizations. Follow-up requests for assistance in subsequent stages of the development cycle of artificial intelligence applications are common and encouraged.

### Building capacity

#### Use best practices from software engineering

Research projects are “routinely unique” by nature and often require tailored solutions. While the software industry has developed agile, lean, or other approaches to tackle such projects, these methods have only slowly found their way into research.

Our consulting has assumed organizational tools from these domains such as planning poker, development sprints, and project retrospectives. Fundamental software quality assurance measures like version control, code linting, and coding standards are routinely used.

More advanced approaches like reproducible environments and workflows, continuous testing, integration, and deployment pose immediate challenges to the data science domain on a day-to-day basis, ranging from the variability of data modalities and the skillsets of involved scientists to the swift integration of tests on high performance computing hardware. Those practices become integral parts of scientific projects and hence raise their quality and diffuse into the scientific communities.

#### Attract, and retain, qualified staff

We also rely on a great freedom and flexibility on the job to attract staff. The job title, and the consulting experience, are perceived as assets for future careers in industry. And indeed, some consultants left after a few years and several successful projects, mostly attracted by higher salaries in industry or for personal reasons.

Just like in industry consulting, the variety of projects and topics spanned in a short time permitted us to attract top talents. Our unique selling point is perceived as we advance scientific knowledge. It is not clear yet if consultants will be able to pursue successful careers in academia, as they are not incentivized to publish first author publications, and the consulting projects do not create a consistent research portfolio. With 3 years of hindsight, we cannot estimate how long we will typically retain our employees, but a large majority have already wished to have their contracts extended beyond 2 years.

#### Stimulate internal communication

A key feature of Helmholtz AI is the network of six consultant teams, spread across Germany and focusing on different research fields (Figure 1A). Our network was set up during the COVID-19 pandemic, and a majority of our staff was recruited only after the outbreak. On one hand, the lack of personal communication posed a significant challenge in creating a team spirit. On the other hand, confined to working from home, colleagues in different institutions felt almost as close as their own team members.

Despite the limited success of online group formats, outstandingly positive point-to-point communication was possible on many occasions. The required team solidarity on a national level improved greatly after the first in-person retreat, during which, in dedicated sessions, the consultants began a more vivid exchange.

Following up on this, biyearly in-person consultant retreats proved to be a good rhythm to federate this network. Just as this comment reflects the success factors of the concept, the team members highly appreciate the reflection with peers on best practices in AI consulting.

### Having an impact

#### Foster knowledge transfer

The main success of our setup is to create knowledge transfer from the expert consultant to the field researcher throughout the collaboration, which is acknowledged in our survey (Figure 1C). Interestingly, knowledge transfer additionally happens on other levels.

First, as a result of the in-depth collaboration with diverse scientific projects, the consultant teams are acquiring a pool of knowledge that efficiently transfers between projects.

Second, the match between consultants and projects is based on methodological expertise, and matching of several consultants from different teams is encouraged. In consequence, consulting projects are likely to span across research fields. We indeed report over 40 interdisciplinary papers over the course of 3 years, illustrating the effective transfer of methods between fields, with high impact.

#### Scale efficiently and sustainably

On-demand consulting through dedicated collaborative projects does not scale up efficiently. And demand is increasingly exceeding capacity. A streamlined selection process, including formal and scientific criteria is therefore being put into place. But most importantly, through our experience, we have obtained a unique perspective on what researchers want and need, and our structure is adaptable. To scale up our impact, we are therefore setting up a coordinated teaching offer targeted at this post-graduate audience and aimed at complementing the existing offer through dedicated courses.

We are piloting consulting for ethics in AI development, starting with health applications. We are also increasingly encouraging co-supervision of students by the consultants, leaving implementation tasks on the collaborators’ side whenever possible and thereby training the supervisor to supervise such projects as well. Finally, we are starting to offer one-on-one consulting sessions and support for code reproducibility to efficiently guide researchers who have embarked themselves in an AI project, on an ad hoc basis.

#### Design specific KPIs

Measuring the success of such a large-scale initiative is, especially for funding agencies, a crucial step in process management. Unlike classical research, however, typical key performance indicators (KPIs), such as number of publications, impact factors or citations, apply only to a limited degree to our consulting approach. To evaluate the breadth of our activities, we added specifically designed KPIs to include the number of requests processed; the number of workshops, trainings, and community events held; and the number of resources published (including software tools, datasets, and tutorials).

We are also tracking publications and grant applications submitted as a direct result of consulting that are brought to our knowledge. But our activities also have a great, more indirect, impact on the education and training of the research community. We therefore established an end-of-collaboration survey (Figure 1C) to monitor how knowledge is efficiently and sustainably transferred to our partners. This could be used to craft further KPIs that would help evaluate our impact.

Beyond our network, this reflection could help advance the design of new KPIs in academia, better reflecting the variety of ways in which research impacts scientific knowledge and society.

### Conclusion

In order to give a head start to researchers, like our persona Anna, at adopting AI in their research, we have established an in-house consulting for an audience of 23,000 scientists. While this concept is common in larger commercial companies, only a few research organizations (Netherlands Escience Center, INRIA, and Software Sustainability Institute) have attempted it so far at scale—if at all.

Helmholtz AI consulting was established as an infrastructure, which relies on a sustainable organizational structure and is accessible to everybody. It employs both a streamlined and flexible process to cater to academic demand. Moreover, it has matured to a broad and diverse network to push science at Helmholtz to new limits using AI.

In the setup phase, we have used best practices from consulting and software engineering and adapted them to the academic setting. In this process, we gained a unique perspective on the researchers’ needs that will enable us to scale our activities. We believe that our model has a prototypical value and the potential to increase the dynamics in the scientific endeavor of the Helmholtz association.

## References

[1] Jeong Y., de Andrade E Sousa L.B., Thalmeier D., Toth R., Ganslmeier M., Breuer K., Plass C., Lutsik P. (2022). Systematic evaluation of cell-type deconvolution pipelines for sequencing-based bulk dna methylomes. Brief. Bioinform..

[2] Toker A., Kondmann L., Weber M., Eisenberger M., Camero A., Hu J., Pregel Hoderlein A., Şenaras Ç., Davis T., unknown (2022).

[3] Pargmann M., Ebert J., Kesselheim S., Maldonado Quinto D., Pitz-Paal R. (2022). https://elib.dlr.de/193816/.

[4] Starke S., Thalmeier D., Steinbach P., Piraud M. (2022).

[5] Asgarimehr M., Arnold C., Weigel T., Ruf C., Wickert J. (2022). Gnss reflectometry global ocean wind speed using deep learning: Development and assessment of cygnssnet. Remote Sensing of Environment.

[6] Weiel M., Götz M., Klein A., Coquelin D., Floca R., Schug A. (2021). Dynamic particle swarm optimization of biomolecular simulation parameters with flexible objective functions. Nat. Mach. Intell..

[7] Wirth R., Hipp J., Mackin N., Company P.A., Ltd P.A.C., Mackin N., Company P.A., Ltd P.A.C. (2000).

